# Western listeners detect boundary hierarchy in Indian music: a segmentation study

**DOI:** 10.1038/s41598-021-82629-y

**Published:** 2021-02-04

**Authors:** Tudor Popescu, Richard Widdess, Martin Rohrmeier

**Affiliations:** 1 Department of Behavioural and Cognitive Biology, Universität Wien, Althanstrasse 14, 1090 Vienna, Austria; 2 Medizinische Universität Wien, Spitalgasse 23, 1090 Vienna, Austria; 3 Department of Music, School of Arts, SOAS University of London, London, UK; 4 Centre for Music and Science, École Polytechnique Fédérale de Lausanne (EPFL), Lausanne, Switzerland

**Keywords:** Psychology, Human behaviour

## Abstract

How are listeners able to follow and enjoy complex pieces of music? Several theoretical frameworks suggest links between the process of listening and the formal structure of music, involving a division of the musical surface into structural units at multiple hierarchical levels. Whether boundaries between structural units are perceivable to listeners unfamiliar with the style, and are identified congruently between naïve listeners and experts, remains unclear. Here, we focused on the case of Indian music, and asked 65 Western listeners (of mixed levels of musical training; most unfamiliar with Indian music) to intuitively segment into phrases a recording of sitar ālāp of two different rāga-modes. Each recording was also segmented by two experts, who identified boundary regions at section and phrase levels. Participant- and region-wise scores were computed on the basis of "clicks" inside or outside boundary regions (hits/false alarms), inserted earlier or later within those regions (high/low "promptness"). We found substantial agreement—expressed as hit rates and click densities—among participants, and between participants' and experts' segmentations. The agreement and promptness scores differed between participants, levels, and recordings. We found no effect of musical training, but detected real-time awareness of grouping completion and boundary hierarchy. The findings may potentially be explained by underlying general bottom-up processes, implicit learning of structural relationships, cross-cultural musical similarities, or universal cognitive capacities.

## Introduction

Everyday experience listening to music suggests that even music in unfamiliar styles appears to "make sense" to some degree, as opposed to sounding random. It is plausible that listeners are cognitively able to perceive structural features of music in real time, as the music unfolds; or, to put it differently, that their active perceptual organisation of the auditory stimulus takes account of features that reflect the composer’s or performer’s structural intentions. One aspect to which listeners may be sensitive is the hierarchical grouping of musical events (into so-called "sections", "phrases", "motifs" etc.) and the boundaries between such groups. It is not clear, however, how far this aspect of music perception depends on familiarity with the musical style, cross-cultural musical features or cues, or universal cognitive capacities; nor to what degree boundary perception is based on "deep" musical structure (implying non-local, long-range musical relations) and/or on "surface" cues.

Segmentation, and the perception of hierarchical grouping structure in music, are the subject of many theoretical and empirical studies, mostly of the tonal styles familiar to Western readers^1–5^. Notably among theoretical studies, Lerdahl and Jackendoff’s Generative Theory of Tonal Music (GTTM)^6^ models Western listeners' musical intuitions by proposing explicit rules whereby listeners are presumed to infer certain cognitive structures, specifically including hierarchical grouping structure, from the musical surface of a Western tonal piece. In empirical studies, patterns exhibited by listeners in performing tasks of phrase segmentation in music have been linked to musical tension^7^, tonality vs atonality^8^, and speech prosody^9^. Additionally, they have been found to be modulated by musical expertise^1,10^.

It cannot, however, be assumed that the same rules and principles govern grouping structure in music outside the Western canon, without empirical verification. Nor, on the other hand, can it be assumed that the perception of grouping boundaries depends on familiarity with a given musical style, whether this familiarity is acquired through enculturation (that is, immersion in the culture in question) or in other ways, such as listening to recordings. These questions can be investigated by studying the responses of participants to music with which they are culturally unfamiliar^11^. This approach allows disambiguation of culture-dependent vs -independent aspects of music perception and cognition, and potentially contributes to a better understanding of human cognitive capacities and proposed universal features of music^11,12^. While the existence of musical universals has been doubted in ethnomusicology for some decades^13^, it has recently been the subject of renewed interest in relation to the origins and evolution of human music^14–17^.

Despite the potential of this approach, only a small minority of empirical segmentation studies so far have sought a cross-cultural perspective, with the aim of comparing level of agreement between participant groups familiar and unfamiliar with the music in question. Ayari and McAdams^18^ compared segmentation of Arabic improvised instrumental music (*taqsīm*) by listeners of European and Arabic cultural origins. Listeners of both groups agreed on boundaries featuring salient surface features such as pauses and register changes, but listeners of Arabic origin also made segmentations defined by subtle modal changes that went unnoticed by the Europeans. Similar findings based on Arabic music are reported by Lartillot and Ayari^19^, who also compared listener responses with computational models, and such evidence suggests that while cultural familiarity with a musical style confers some advantage, listeners without such familiarity also perceive elements of grouping structure.

Such group comparisons have also been made in terms of neuronal responses obtained during segmentation tasks. Nan et al.^20,21^ found neurophysiological evidence (in the form of event-related potentials, ERPs) that melodic phrase boundary perception in Chinese and Western melodies, by Chinese and German listeners, was influenced both by cultural familiarity with the style and by surface features (silences between phrases). Nan et al.^22^ attribute the different neuronal responses elicited by culturally-unfamiliar music to the higher demands it places on attention systems and auditory processing.

Despite this difference in neuronal responses, empirical studies suggest that listeners unfamiliar with a certain musical style can sometimes exhibit striking agreement with those who are enculturated to it. Mungan et al.^23^ studied segmentations of Turkish music by Turkish musicians, Turkish non-musicians, and Western listeners, and compared the results with segmentations by two "makam music experts". The authors hypothesised that "if online segmentations are driven mostly by *surface features*, i.e., bottom-up processes, we should observe considerable overlaps within and across all three groups [regardless of musicianship or enculturation]"^23^; whereas if segmentation were dependent on implicit or explicit culture-specific schemata, one would expect different groups of listeners to perform differently. The authors go on to report extensive agreement between all groups of listeners, which they therefore attribute mainly to bottom-up processes and Gestalt-type grouping features, namely pitch, contour and durational separation. In another study, evidence for listener sensitivity to features of music in an unfamiliar musical system similarly extends to musically-induced emotion, correctly identified in Indian music by a sample of listeners unfamiliar with the style in a manner predicted by surface cues such as tempo, rhythmic complexity, melodic complexity, and pitch range^24^. The evidence of this and other cross-cultural studies compellingly refutes an earlier hypothesis: that Western listeners cannot hear music in an unfamiliar style, such as Indian music, "as music", on the grounds that perception of its structure and emotional connotations depends entirely on enculturation^25^.

The available cross-cultural segmentation studies employ a variety of methodologies and musical materials. Musical stimuli include both improvised performances^18,19^, and score-based, synthesised realisations of composed items^20–23^. Although segmentation studies based on Western music frequently compare the performance of musically trained and untrained listeners^1,10,23,26–28^, among cross-cultural studies only Mungan et al.^23^ compare (Turkish) musicians and non-musicians, finding higher convergence of the musicians with the expert listeners. Another variable is the number of times the listeners hear and segment the piece; Mungan et al. report that in three segmentation trials, listeners were already relatively accurate in the first trial, with little subsequent change^23^.

Different studies also investigate different types of response. While ERP investigations focus on very rapid, involuntary responses^20–22^, empirical experiments take into account slower, voluntary responses, and adopt a variable degree of tolerance for delayed reactions. Some allow retrospective identification of boundaries in the light of subsequent musical changes—for example, changes in mode, key, timbre, or rhythm; such retrospective responses are indicated by marking a written score^18,29^. It is important to note that segmentation responses that are given before or after the start of the next segment indicate different perceptions: perception of completion, in the former case, or of initiation (combined with completion), in the latter. In the present study, we focus exclusively on the former type, that is, evidence for real-time awareness of grouping *completion*, irrespective of subsequent changes. This approach is appropriate to the musical style concerned*,* in which abrupt changes do not occur: the elaboration of the melody across the pitch-range of the instrument unfolds very gradually, without changes of mode, key, timbre, or rhythm.

In the present study, participants were set a segmentation task while listening to a recording of North Indian classical music, in the *ālāp* style (see “Materials”). In this article, we focus on the following questions:

1. To what degree are Western listeners able to identify phrase boundaries in Indian music, despite having no prior experience or training in that music? Is there enough information in the musical signal alone to afford boundary perception in listeners who are not familiar with the style?
2. How far do our listeners segment music with reference to "surface cues" (e.g. acoustic features implying bottom-up processing) or to "deep structure" (implying long-range musical relationships presumably processed top-down)?
3. How far does (Western) musical training (as defined in “Participants”) predict how a listener performs during a segmentation task of unfamiliar music? Do musically trained listeners exhibit greater awareness of "deep structure" as defined above?

We approached these questions with the corresponding hypotheses:

1. That the active process of perceptual organisation would lead our listeners to infer segment boundaries; and that if their inferences reflected features of the auditory stimulus in a consistent way, their responses would converge significantly across participants, and with expert segmentations.
2. That listeners might exhibit evidence of awareness of long-range structural processes, by (a) responding more to higher levels of grouping hierarchy than to lower ones, and/or (b) responding relatively promptly to the arrival of a boundary, suggesting that they expected it.
3. That responses by musically trained participants might agree more closely with the expert segmentation, and/or that they might show more awareness of structure in the manner defined in hypothesis 2, as compared with untrained participants.

## Methods

### Participants

This study constitutes an extensive statistical reanalysis of the dataset published in Ref.^30^. Sixty-five UK-based adults of mixed musical background took part in the experiment (for demographic information, see Table 1). All were students at the University of Cambridge or at SOAS University of London, and were mostly from a Western cultural background. Six participants declared themselves familiar with Indian music; their data points are highlighted in subsequent plots. Approximately two thirds of the sample had some training, namely in Western music (for details, see “Procedure”).

**Table 1:** Demographic information for the participant sample. Years/hours are indicated as range and mean ± SD. MD indicates missing (unavailable) data.

		*Toṛī*	*Multānī*	Total
Sample	N	32	33	65
	Male	MD	16	
	Female	MD	17	
	Ages (years)	MD 24.40 ± MD	19 – 58 24.27 ± 7.25	
Musical training	N reports	31	30	61
	Instrument training (years)	0–20 7.60 ± 6.82	0–20 9.53 ± 6.27	
	Weekly practice (hours)	0–30 6.39 ± 7.64	0–25 5.80 ± 6.14	

### Materials

The two stimuli consisted of a pre-recorded, c. 5-min long *ālāp* in each of two melodic modes (*rāgas*), Toṛī (also spelled Toḍī or Todi) and Multānī. Both ālāps were performed by a professional sitarist, Dharambir Singh (henceforth DS), who was unaware of the purpose and design of the experiment. Details of the structure and melodic features of the rāgas can be found elsewhere^30–33^. Briefly, an *ālāp* is an improvised exposition of a rāga, introducing the notes and melodic motifs of the rāga in a systematic manner. The performer starts from scale degree $\widehat{1}$, then introduces successively higher pitches and motifs until $\widehat{1{^{\prime}}}$ or some higher degree is reached. Periodically during this process, and at the very end, the performer returns to the starting-point, $\widehat{1}$^34^. In our recordings the octave below $\widehat{1}$ was also briefly explored at the beginning of each ālāp (see example in Figure S1 in the Supplementary Materials).

*Ālāp* is performed in apparently free, non-metrical rhythm, with no rhythmic accompaniment; any pulse or "beat" present in the performer's mind is hardly apparent to the listener and is not consistently grouped into larger metrical units^35^. Consequently, grouping cannot be predicted on grounds of pulse or metre, as it can in metrical music. In sitar playing, the drone strings are plucked frequently, between groups of 1–5 melodic pitches in our examples; this is likely to affect grouping perception only at very superficial hierarchical levels.

*Ālāp* is the first of a sequence of sections in different styles, including sections that are partly and wholly metrical, improvised and pre-composed, solo and accompanied, through which a *rāga* is conventionally rendered in concert performance. *Ālāp* is not precomposed but improvised, so no prescriptive scores exist that could be used as a basis for synthesised stimuli as in^23^. Given the complexity and flexibility of both pitch and rhythm in ālāp, digital synthesis would be an unfeasible and unrealistic substitute for the norm of live performance. We therefore used improvised studio performances recorded for us by the sitarist, on grounds of both feasibility and ecological validity (cf^18,19^). We chose to present two rāgas in order to explore how far the perception of segmentation generalises across rāgas. Both rāgas employ the same basic scale (*Toṛī ṭhāṭ*), chosen to be relatively unfamiliar to Western listeners: 1 b2 b3 #4 5 b6 7.

The term *rāga* denotes the underlying modal schema of which an *ālāp* performance is a token or exemplar. For convenience, we use "rāga" and the names Toṛī and Multānī to denote the two ālāp recordings used in our study, since *rāga* is the principal distinction between them. Where it is necessary to refer to the underlying modal schema as such, we use "rāga-mode".

### Procedure

Each participant was randomly assigned to one of two groups, corresponding to the rāgas Toṛī and Multānī. Self-reported number of years of instrument training and weekly number of hours of practice were available for most participants (for demographic information, see Table 1). The two groups did not significantly differ in either of those measures (independent samples t tests: *t*(62) = 1.15 and *t*(62) = 0.33 respectively; both n.s. at *p* > 0.1). The two measures were each standardised into *z* scores within each group; the average of those two *z* scores was operationalised as the musical training (henceforth: musicianship) score.

The experiment was presented and responses captured via a Flash ActionScript file running on a PC. Before the segmentation task was introduced, participants heard a short example of ālāp in a rāga (Jaijaivantī) unrelated to Toṛī and Multānī, to demonstrate the melodic and rhythmic style of ālāp, but they were not required to make any response. This example was not used subsequently in the experiment. Participants were then told that they would hear another example, during which they would be asked to press the spacebar on a PC keyboard (henceforth "click"), "whenever you think a phrase within the melody ends". Since the aim was to elicit intuitive, spontaneous responses to the task, the term "phrase" was not further defined, and participants were asked to follow their intuition. There was then a short practice session, during which participants heard the first 45 s. of the assigned ālāp and could try out pressing the spacebar to indicate their responses. This practice session was not repeated.

Each participant then listened to their assigned ālāp. The duration of the ālāp was represented on screen as a horizontal black line, along which a black marker moved from left to right as playback progressed. Pressing the spacebar generated a red marker at the appropriate point along the same line, and this time-point relative to the duration of the ālāp was automatically recorded. There was no restriction on the number of boundaries that a participant could insert, and no opportunity to revise (move, delete, add retrospectively) boundaries. As soon as the task had been completed, it was repeated, exactly as before, but without the practice session; during this second hearing participants could no longer see their responses from the first hearing.

### Expert segmentation

Prior to analysing the segmentation data, one of the authors (RW), a specialist in Indian music theory, carried out a transcription of both ālāps, including segmentation into phrases and sections. Subsequently, expert segmentation data was also obtained from the performer (DS), for cross-validation purposes. The two segmentations were found to be largely in agreement: every boundary identified by author RW was also considered a boundary by performer DS, and there was a 77% overlap in choice of level. The main differences concern additional "Level 3" boundaries marked by the performer, which we did not take into account. Modification of RW's segmentation was therefore deemed unnecessary (see section S1.3 in the Supplementary Materials for details).

By "expert-defined boundary *region*" (EDBR) between two consecutive phrases, we refer to the time interval between the onset of the last melodic pitch of phrase n, and the first of phrase n + 1. Any plucks on the drone-strings of the sitar during this interval were ignored. This definition allows time for participants to hear the last note of phrase n, and prepare and execute a response, before the start of phrase n + 1. These "boundary regions" are of variable length, at the discretion of the performer.

We distinguish a two-level hierarchy of EDBRs, Level 1 ("Sections") and Level 2 ("Phrases"). Level 1 boundaries mark the ends of longer sections of the performance defined by melodic returns to the scale-degree 1 (at any of three octave positions), as predicted by the conventional structure of an ālāp. Level 2 boundaries mark shorter groupings of pitches (i.e. phrases) within a section, sometimes ending on pitches other than 1; in defining this EDBR level, the duration of final pitches and the subsequent pauses, and the melodic grammar and conventional motifs of each rāga-mode were taken into account. Thus the EDBRs reflect both top-down schematic structural knowledge, and bottom-up processing of surface features. A Level 1 boundary is de facto also a Level 2 boundary, but not vice-versa. For the ālāp in rāga Toṛī, five Level 1 and thirteen Level 2 boundaries were identified; and for Multānī, six Level 1 and twenty-eight Level 2. Segmentation at further levels is also possible, e.g. sub-phrases within phrases (see section S1.3). For present purposes, however, we refer to Levels 1 and 2 only; we believe that these levels can account for the majority of our participants' responses.

Nevertheless, our segmentation is not a definitive or complete analysis of the grouping structure (see section S1.3 for a different but compatible analysis). Therefore, our conventional designation of participant responses as "correct" or "incorrect" does not refer to objective correctness, but merely enables us to analyse the extent of convergence between listeners and experts. However, the extensive agreement between our segmentation and that of the performer (see S1.3) indicates a high degree of "emic" correctness.

The example in Figure S1 shows our segmentation of the beginning of the ālāp in Multānī, illustrating the relationship of Level 1 and Level 2 EDBRs.

### Analyses

All data analyses and plots were performed in MATLAB (Mathworks Inc.; Natick, MA).

#### Scoring

Following signal detection theory^36^, a click was considered a "hit" if it occurred within the time span of an EDBR. We assumed that each "hit" represents the listener's response to the encompassing EDBR, rather than a delayed or anticipatory response to an earlier or later EDBR. Furthermore, while clicks falling immediately before or after the EDBR could be interpreted as anticipations or delayed responses respectively, these were not counted as "hits". Instead, a click was considered a "false alarm" if it occurred in "interstitial" space, i.e. the out-of-boundary space between EDBRs. For hits, the timing of a participant's click relative to the EDBR onset was measured and analysed as the "promptness" of the response (see “Promptness” below).

#### Signal detection theory measures

To compare listeners' ability to identify different hierarchical grouping levels, we computed *hit-* and *false alarm* rates (HR, FAR), defined as the number of hits or false alarms respectively, divided by the total number of "signal present" or "signal absent" trials (EDBR and non-EDBR periods respectively; see section S1.5 for details). HR and FAR were computed participant-wise, averaging across the two listenings, separately for Level 1 (section-level) and Level 2 (phrase-level) EDBRs. For a more detailed description of these measures, see section S1.5.

Based on these rates, we also quantified a participant's general ability to identify phrase boundaries throughout the ālāp (i.e., clicking inside EDBRs at any level while refraining from clicking outside of them) using the *sensitivity index*, defined conventionally^37^ as

$${\text{d}}^{\prime} = {\text{z}}\left( {{\text{HR}}} \right) - {\text{z}}\left( {{\text{FAR}}} \right).$$

#### Promptness

For each participant we quantified—for each hit, in each listening repetition—how promptly they detected the end of the current phrase. For this we defined, for any given hit, a "promptness" score that ranged between 1 (if the click occurred at the very beginning of the EDBR, i.e. a prompt response) and 0 (if it occurred at the very end, a delayed response; see Figure S2). We computed a scoring function based either on linear (a*x + b) or a reciprocal function (c/*x*), with values in the 0–1 range. These both yielded similar results, and we therefore retained the linear definition. Only the first click within each EDBR was used for promptness scoring.

To check whether participants responded more promptly to Level 1 than to Level 2 boundaries or vice-versa, promptness scores were summed across hits of EDBRs at each level separately, and normalised (divided) by the corresponding number of EDBRs in the ālāp. This led to cumulative scores for section and phrase awareness for each participant at Levels 1 and 2 respectively.

#### Repetition (listening) number

All analyses were performed on the combination (average or sum) of repetitions (listenings) #1 and #2; this is because the differences between responses to the two listenings were small on all relevant measures. In all analyses, we used the median rather than the mean as a measure of central tendency, given its robustness to outliers (using means yielded similar results in this case). For correlations between participant-wise performance measures (HR, FAR, *d'* and promptness) across listenings, please see Figure S6 (all these correlations were above *r*s > 0.58).

## Results

### Click clustering and convergence with EDBRs

Table 2 summarises the number of clicks inserted by participants at each repetition, as well as the number of EDBRs defined and their duration.

**Table 2:** Descriptive statistics regarding the number of inserted clicks, and number and duration of defined EDBRs.

		*Toṛī*	*Multānī*
Number of clicks across participants	Rep. 1	1–38 17.8 ± 9.3	7–46 21.3 ± 9.9
	Rep. 2	0–43 17.5 ± 10.8	6–51 21.7 ± 10.8
Number of defined EDBRs	Level 1	6	7
	Level 2	13	27
	TOTAL	19	34
Durations of EDBRs (seconds)	Level 1	1.8–10.8 s 5.4 ± 3.1 s	1.3–7.8 s 4.2 ± 2.4 s
	Level 2	1.9–7.3 s 3.4 ± 1.5 s	1.3–4.8 s 2.5 ± 0.9 s

Number of clicks and EDBR durations are indicated as range and mean ± SD. *Rep*.,  repetition (listening) number.

**Figure 1:**
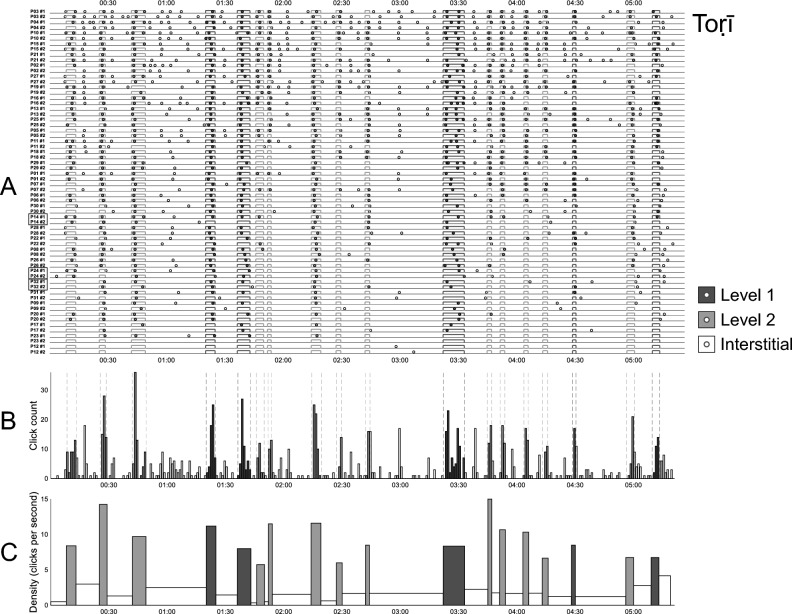

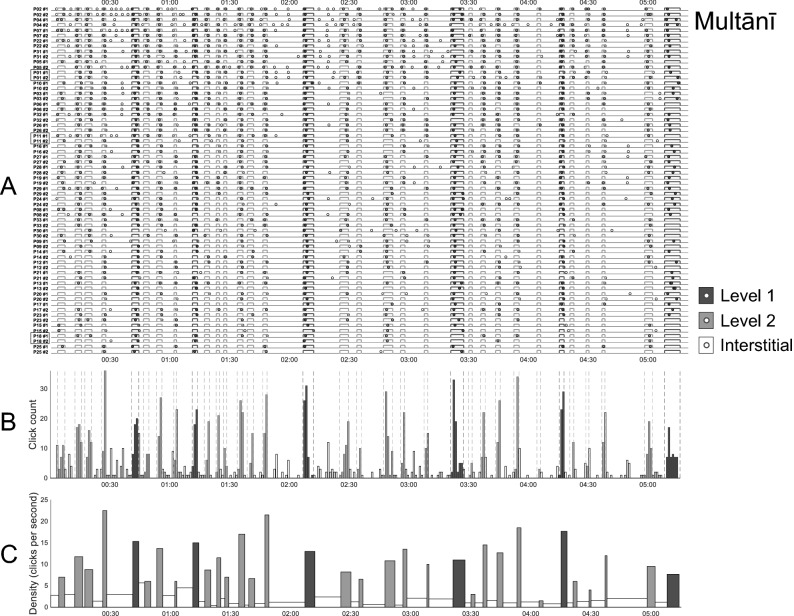
Participant-inserted clicks in relation to EDBRs. For each rāga, top-panel plots (**A**) depict, on each line, the occurrence of clicks (dots) and EDBRs (rectangles) along the timespan of each of a participant's ("P") two listenings ("#1" and "#2"). Participants are listed in decreasing order of their total number of clicks across repetitions. Middle-panel plots (**B**): histograms counting clicks across all participants and across both listenings, within 1 s bins; vertical dotted lines delimit EDBRs. Bottom-panel plots (**C**): click density histograms at region level; counts are summed for each region and normalised (divided) by the region's duration. Level 1 boundaries are represented in dark grey, Level 2 in light grey; circles or bars thus filled represent hits (within-boundary clicks), while hollow (white) regions represent false alarms (interstitial clicks). Participants familiar with Indian music are highlighted in black boxes. For animated versions of these plots, with a cursor moving in time with the music, please see the video files uploaded to OSF. https://osf.io/khvmf/.

In Fig. 1, panels A depict the raw *distribution* of clicks for the two rāgas, across all participants and across both within-participant listenings. The click distributions are summarised in the histograms below them (panels B) which count clicks within 1-s bins. A high degree of clustering of participants' responses is clearly visible: the histograms have distinct peaks and valleys, with clicks that tend to cluster in particular regions that converge well with EDBRs. Interstitial regions (between EDBRs) are underpopulated by comparison, despite occasional "false alarm" peaks.

Further insight can be gained by partitioning each rāga's time course into contiguous time regions, corresponding either to the EDBRs or to the interstitial regions between them. Panels **C** of these figures depict region-level, time-normalised histograms, in which bar height for a given region represents the total number of clicks occurring within that region, divided by the region's duration. These histograms thus depict the *density* of clicks per second in each region, where greater density denotes higher inter-participant agreement. It can be observed how the densities within EDBR regions (whether of Level 1 or 2), depicted in panels C by filled rectangles (dark and light grey respectively), are consistently higher than those outside them (the interstitial regions: hollow rectangles). These observations are quantified in the next section.

### Effect of boundary level on hit rates and click densities

**Figure 2:**
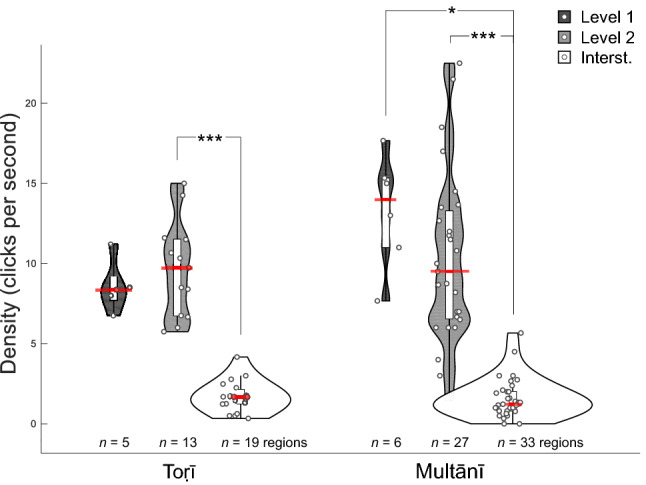
Click densities for each rāga. Dots represent time regions (cf Fig. 1C) and clicks are counted across all participants. Violin plots show click density distributions across all 3 region types: Level 1 (dark grey), Level 2 (light grey) and interstitial (white), extending to the lowest and highest data points in each distribution. Within them are Tukey boxplots, with boxes (white background) drawn between the 1st and 3rd quartiles, representing the IQR (interquartile range), and whiskers extending to ± 1.5 IQR of the box. Red lines indicate medians. As in the remaining figures, asterisks denote level of significance (*p < 0.05; **p < 0.01; ***p < 0.001).

Figure 2 depicts the distribution of click densities across region types (see also Figure S9 for the same measure per-participant). We tested these distributions' deviation from normality to inform subsequent use of parametric vs non-parametric statistics. Collapsing across region type, both distributions violated the normality assumption (one-sample Kolmogorov–Smirnov test: *p*s < 0.0001), thus the effect of region type was tested with the Kruskal–Wallis test, used as a non-parametric 1-way analysis of variance. The latter test was significant both for Toṛī (*χ*^2^ = 27.11, *η*^2^ = 0.74, *df* = 2, *p* < 0.0001) and for Multānī (*χ*^2^ = 46.42, *η*^2^ = 0.71, *df* = 2, *p* < 0.0001). Follow-up tests using the Wilcoxon signed rank test revealed significant or close-to-significant differences between the interstitial and each of the two EDBR regions in each rāga (statistics in Table 3). The difference between Levels 1 and 2 is not statistically significant in either rāga. Comparing rāgas for each region type revealed non-significant differences, albeit with a *p* value approaching significance for Multānī's descriptively-higher Level 1 densities (Wilcoxon rank sum statistic = 20, *p* = 0.082).

**Table 3:** The Wilcoxon signed rank test statistic and its *p* value, for pairwise comparisons of click densities between region types.

Contrast	*Toṛī*		*Multānī*	
	Signed rank	*p*	Signed rank	*p*
Level 1 > Interst	15	0.062	21	0.031
Level 2 > Interst	91	< 0.001	378	< 0.0001
Level 1 > Level 2	5	0.625	13	0.687

**Figure 3:**
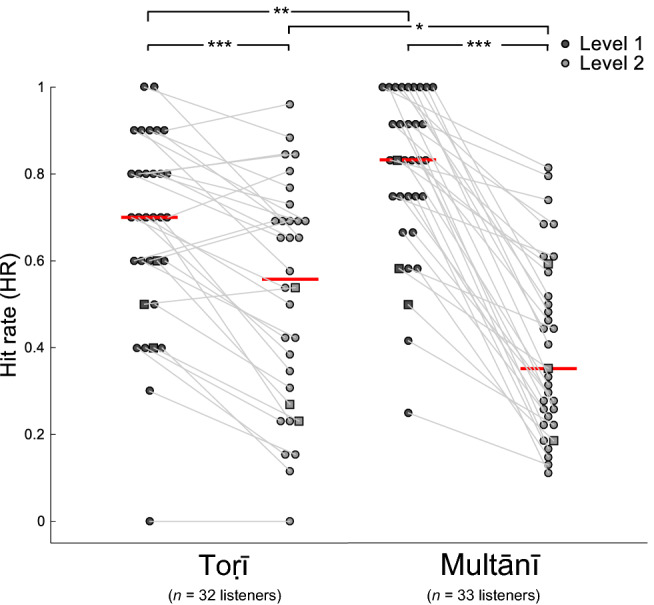
Hit rates (HRs) for Toṛī (left) and Multānī (right). Unlike Fig. 2, dots here represent participants, i.e. their HRs, for Level 1 (black) and Level 2 (grey) EDBRs, averaged across listenings, and connected for each participant by thin grey lines. Red horizontal lines represent each category's median. Squares represent participants familiar with Indian music (*n* = 3 in each rāga).

HR distributions, for Level 1 and Level 2 EDBRs, are depicted in Fig. 3. As with click densities, these deviated from normality (one-sample Kolmogorov–Smirnov test: *p* < 0.0001, for both rāgas), thus the effect of region type was tested with the Kruskal–Wallis test, significant both for Toṛī (*χ*^2^ = 6.09, *η*^2^ = 0.08, *df* = 1, *p* = 0.014) and for Multānī (*χ*^2^ = 33.58, *η*^2^ = 0.51, *df* = 1, *p* < 0.0001). Follow-up tests using the Wilcoxon signed rank test revealed significant differences between levels both for Toṛī (signed rank = 465, *p* < 0.0001) and for Multānī (signed rank = 561, *p* < 0.0001).

In each rāga, HRs at Level 1 are significantly higher than for Level 2. Comparing rāgas at each level revealed significantly greater HRs for Multānī compared to Toṛī at Level 1 (Wilcoxon rank sum statistic = 846, *p* = 0.006), but smaller HRs for Multānī compared to Toṛī at Level 2 (Wilcoxon rank sum statistic = 1207, *p* = 0.047).

While the density results in Fig. 2 indicate that participants responded more to EDBRs than to Interstitial regions, but do not indicate a consistent significant difference in response to Level 1 than Level 2 EDBRs, the Hit Rate results in Fig. 3 indicate significantly greater response to Level 1 than to Level 2, in both rāgas. This latter effect is more marked in Multānī than Toṛī, which perhaps confirms the similar trend observed in Fig. 2. That is, there is evidence that section boundaries were perceptually more salient than phrase boundaries, especially in Multānī.

According to Fig. 3, Level 1 recognition was superior in the Multānī group to that of the Toṛī group, but the opposite held for Level 2. Such differences may be due to differences in modal structure (see “Local cues or structural awareness?” section of Discussion).

### Promptness scores

**Figure 4:**
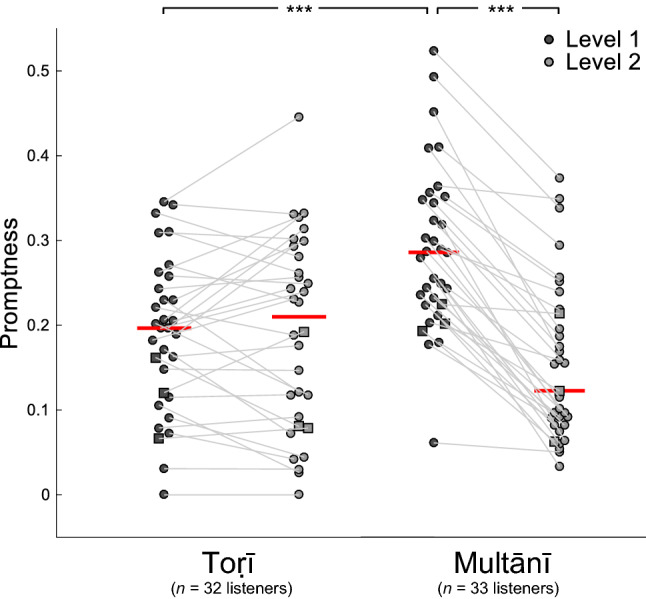
Promptness scores for Level 1 and 2 EDBRs, in each rāga. Dots represent individual participants' promptness scores, averaged across listenings, cumulated across hits and normalised by the corresponding number of EDBRs in each case. Squares represent participants familiar with Indian music (*n* = 3 in each rāga).

Promptness scores (see “Analyses”), cumulated across hits and normalised by the corresponding number of EDBRs in each ālāp, show a wide range of values (Fig. 4), indicating a diversity of response styles: participants who click early in the EDBR (high promptness) are likely to have foreseen the phrase-end, whereas those who click late (low promptness) may do so because there has been an interval of time since the last melodic pitch onset.

Despite the wide range of promptness scores, there is a rather even distribution, providing no grounds for separating "structural listeners" as a group from "gap listeners"—as a bimodal distribution would have suggested (Hartigan's Dip Test for unimodality: all *p* values > 0.47).

A *rāga* × *level* mixed-effects factorial ANOVA was conducted, which revealed a significant main effect of *level* (*F*(1,126) = 65.94, *p* < 0.0001, *η*_p_^2^ = 0.511 with 95%CI [0.372, 0.658]) and a significant interaction (*F*(1,126) = 72.51, *p* < 0.0001, *η*_p_^2^ = 0.536 with 95%CI [0.404, 0.669]). Post-hoc tests done to decompose this interaction revealed, for Multānī but not for Toṛī, higher promptness at Level 1 than at Level 2 (*F*(1,126) = 140.53, *p* < 0.0001, *η*_p_^2^ = 0.691 with 95%CI [0.594, 0.788]). Likewise, at Level 1 (but not at Level 2), promptness was higher for Multānī than for Toṛī (*F*(1,126) = 9.49, *p* < 0.005, *η*_p_^2^ = 0.131 with 95%CI [0.050, 0.245]).

Furthermore, for each rāga, promptness to Level 1 and Level 2 boundaries was significantly correlated across participants (Toṛī: *r*(30) = 0.83, *p* < 0.0001; Multānī: Pearson's *r*(31) = 0.75, *p* < 0.0001; Figure S3). This suggests that they reacted with a consistent degree of promptness to both cue types.

Finally, Figure S7 depicts the correlation between HR and the average promptness score per hit of each participant. It suggests that, even after correcting for the different number of hits between participants, a listener's average promptness per hit can (at least for Toṛī) still be predicted by their hit rate, implying that listeners to Toṛī who identified EDBRs more consistently also did so more promptly. Lending validity to this result, the same correlation for FAR is n.s. (Figure S8).

### Effect of musicianship

Contrary to what might have been expected, the cross-participant correlation between musicianship score and the sensitivity index *d'* was not significant, for either rāga (both *p* values > 0.3; see Figure S4). Also non-significant were the cross-participants correlations between musicianship and promptness, for both types of boundary and for both rāgas (all *p* values > 0.14; see Figure S5), and between musicianship and the hit- and false-alarm rates (all *p* values > 0.44). Thus, participants with musical training were not consistently in greater agreement with the expert segmentation, and were not consistently more prompt in their responses, than those without. Evidently musical training in Western music conferred no advantage in a segmentation task involving culturally unfamiliar music.

## Discussion

The purpose of our study was to investigate the responses of Western listeners to a musical style with which they were unfamiliar, with regard to segmentation: that is, the active cognitive process of organising auditory information into groups, separated by boundaries, in real time. In response to our first question, how far listeners are able to segment unfamiliar music, Fig. 1 shows that our participants converged with each other and with expert analyses to a high degree. Figure 2 further shows that click densities in expert-defined boundary regions are higher than those for interstitial regions. The six participants who were previously familiar with Indian music performed similarly to the other participants: all our main results (that is, the significance and direction of the reported comparisons) remain unchanged qualitatively if these participants are excluded from the analyses.

Secondly, we asked how far segmentation is based on local, surface cues, or on hierarchical structure and non-local processes in the music. We assumed that a higher degree of structural awareness would be indicated by greater responsiveness to higher- than lower-level groupings, and/or by relatively rapid responses to boundaries. We found that participants identified Level 1 boundaries more easily than Level 2 boundaries (Fig. 3), and in the case of Multānī, more promptly (Fig. 4). Listeners to both rāgas clearly identified Level 1 and 2 boundaries as more significant than sub-phrase boundaries ("Level 3" or "interstitial" boundaries). We assumed that those with higher promptness scores could predict phrase ends on the basis of greater structural awareness than those with lower scores, who may have waited to hear a longer "gap" between phrases before responding. Our assumptions seem to be supported by a correlation in one rāga (Toṛī) between hit rate, combined across Levels 1 and 2, and average promptness (Fig. S7). That is, in this rāga at least, those participants who most consistently distinguished EDBRs from interstitial groupings also did so most promptly, and vice versa; suggesting that in this case, greater real-time awareness of structure generated expectations that enabled more rapid responses.

It is in any case certain that all our hit rate and promptness data reflects real-time awareness of grouping completion, irrespective of subsequent musical changes, since we excluded from our definition of "hits" responses made following the end of the EDBR (here our methodology contrasts with those studies where participants were allowed to add or modify boundaries retrospectively, in the light of the following phrase^18,29^).

Thirdly, we asked whether expertise in Western music predicts a participant's performance in the segmentation task. We found no significant advantage conferred by musical training. Neither our index of responsiveness to EDBRs (*d'*) nor the promptness of responses to Level 1 or Level 2 boundaries correlated with musical training.

Our results therefore suggest that, at least in the case of Western listeners to Indian music, listeners unfamiliar with the style can detect phrase boundaries in music to a significant extent, whether they have training in Western music or not. In the following subsections, we consider, first, further evidence regarding question 2; and, second, how our methods and results compare with an earlier cross-cultural segmentation study.

### Local cues or structural awareness?

As noted above, we found some evidence of structural awareness in both groups of listeners, but this evidence differs between groups. This may be due to differences in modal dynamics between the rāgas. Although both rāga-modes use the same scale, in Multānī, the most stable pitch after 1 is 5, which occurs in ascending and descending melodic contexts, and phrase-final and non-phrase-final positions. In Toṛī, by contrast, 5 is eclipsed in importance by b6; it occurs only in descent, and is rarely the final note of a phrase, but moves away to some other point of repose, such as b3 or b6. The listener enculturated to Western music is accustomed to a strong relationship between the "tonic" ($\widehat{1}$) and "dominant" ($\widehat{5}$) scale-degrees; Multānī also exhibits this relationship, and hence this rāga-mode may be easier for such listeners to assimilate, despite the unfamiliar scale. Thus the greater cognitive challenge of Toṛī for Western listeners may explain the more limited awareness of Level 1/2 hierarchy among listeners to the ālāp in this rāga-mode. On the other hand, Fig. 2 and Fig. S7 show that even in this rāga, listeners were aware of the distinction between Level 1/2 boundaries and interstitial articulations.

The question whether listeners identify boundaries on the basis of local discontinuities—especially durational separation between events—or longer-term processes reflecting the larger structure of the music, is often raised in the segmentation literature with reference to the Gestalt theory of grouping principles^19,23,29,38–40^. According to this theory, durational separation or other discontinuities would tend to trigger boundary perception, irrespective of larger structural factors. Here, it is relevant to note that our performer himself exploits this effect, using the degree of durational separation between phrase-final and phrase-initial note onsets to distinguish hierarchical levels of grouping: Level 1 EDBRs are on average longer than Level 2 EDBRs, (see Table 2). Thus an aspect of hierarchical structure is manifested in surface cues that may have influenced participants' responses. However, this can only be the case for participants with low promptness scores: a high promptness score indicates that the participant did not wait to discover how long the inter-onset interval might be before clicking. And as noted above, even participants with low promptness scores clicked *before* the end of each EDBR, as a later response would not be counted by us as a "hit". Thus it seems likely that not only durational separation but also non-local relationships and longer-term processes contribute to boundary perception in this music.

### Comparison with previous results

An initial comparison with earlier cross-cultural and other segmentation studies suggests that our results are similar, despite significant differences of methodology. We take this as further confirmation of our findings.

The study by Mungan et al.^23^ is so far the cross-cultural segmentation study most directly comparable with our own. These authors used materials very different from ours: melodies of Turkish music that were pre-composed, notated, metrical, synthesised and played by machine, whereas our (Indian) melodies were improvised, unwritten, non-metrical, and recorded by a live musician. Their participants included Turkish musicians and non-musicians as well as Western listeners (mostly non-musicians), whereas our listeners were all Western, with a majority (2:1) trained in Western music. Their participants were selected in advance for sensitivity to melodic changes; they were informed about the purpose of the segmentation experiment, and undertook two training tests; the stimuli were played four times, once for familiarisation and three times for segmentation, and participants could see their previous segmentations during subsequent hearings. In contrast, our participants were not pre-selected by ability, were naive to the purpose of the experiment, had limited familiarisation and training in the segmentation task (see “Procedure”), and undertook the task only twice; during the second hearing they could no longer see their responses to the first. Nevertheless, our results and those of Mungan et al. are strikingly similar: as they report, "Overall, we found an astonishing overlap of segmentations not only within each group [of participants] but also across groups. Moreover, segmentations also showed considerable convergence with expert segmentations". In neither study was enculturation necessary to achieve this convergence. Both studies found no significant difference in performance between the participants' two or three attempts at the task.

As in earlier studies (Refs.^19,24^ and especially^29^), Mungan et al. concluded that participants rely primarily on localised surface cues triggering Gestalt effects, especially durational separation between segments. Here our results diverge, with some evidence of awareness of larger processes, as noted above. This pattern could plausibly result, as argued in the *cue-redundancy model* by Balkwill and Thompson^24^, from a combination of psychophysical and culture-specific cues; the latter might themselves arise from culture-specific stereotypes held by unfamiliar listeners, as proposed in the *stereotype theory of emotion in music* (STEM) by Susino and Schubert^41^. Finally, Mungan et al. observe (as do we) that higher-level groupings may be demarcated by surface features (such as durational separation), and that "it may be this lack of a dissociation between music-structural segments and surface-feature-based segments (..) which made it possible for Western listeners to perform so well". In our case, it is the performer who marks the hierarchical structure with shorter and longer gaps between phrases; but as noted above, highly prompt responses do not take account of the full length of a gap, and all hits, as defined by us, occur before the end of a gap.

### Limitations

There are several methodological limitations to our study. First, there was an unavoidable difference in the number of EDBRs between the two ālāps, due to our use of ecologically valid recordings rather than synthesised stimuli. Secondly, we compared real-time segmentation from listeners with a non-real-time expert segmentation. For the latter, transcription, analysis, and multiple re-hearing were employed in order to establish a model segmentation as close as possible to the performer’s likely intentions (as subsequently verified by the performer himself); this task was thus different from that undertaken by the participants. Some previous studies^18,23^ similarly derive the expert segmentation from transcription-based musical analysis, which must also have been made offline, presumably for the same reason. Thirdly, while our study tentatively hints at potential generality by choosing more than one example, more evidence is of course needed for a full cross-cultural claim (which would in any case qualify notions such as that of the "Western" listener^11^). For instance, these results cannot tell us how far the effects generalise to exemplars of Indian music other than the two chosen, and—even less—to other musical traditions^42^. In fact, our results demonstrate that some degree of variation can be expected in responses to different rāgas, owing to their different modal characteristics. Despite this, however, the results from both rāgas exhibit a striking degree of convergence between participants’ responses and expert segmentations (Fig. 1).

### Implications for future research

Given the need for music psychology to adopt a more cross-cultural approach, recently articulated by Jacoby et al.^11^, we hope and expect that more studies will choose to examine more critically factors to do with the listener-music relation that have previously often been implicitly assumed to take on their 'default' Western values. These factors mainly relate to enculturation and familiarity, but also to other topics. Such studies could, for instance, compare different populations in order to address the long-standing question of universals in music perception, and further refine the description of the relative roles of culture and biology in creating e.g. preferences for fundamental (low-level) or emerging (high-level) features of music, such as consonance/dissonance^43,44^ and other phenomena of pitch perception^45^ or production^46^; rhythm^47^; emotion^48,49^ and even of the function of music^14^.

Future research might also investigate what other factors besides durational separation are taken into account in boundary perception. Data such as that obtained in the present study, linked with insights from the performer(s) of the kind provided by our sitarist, would also enable more fine-grained musical analysis, for example of formal and modal structure, improvisatory techniques, and melodic syntax in Indian music, in relation to music perception and cognition. Such analyses could lead to further investigation of hierarchical grouping as a feature shared by music and language.

The generality of the present findings remains to be tested in future studies, which can provide converging evidence by employing a wider variety of samples—e.g. in terms of melodic modes—within the musical style concerned.

## Conclusion

We conclude that our study shows a high degree of culture-independent, real-time awareness of grouping completion on the part of Western listeners unfamiliar with Indian music. We agree with earlier studies that boundary perception is influenced by surface cues, but we also found some evidence of awareness of larger structural processes. We also found that listeners trained in Western music had no significant advantage over non-musician listeners in performing the segmentation task.

In the light of these findings, the question arises: *How* do participants who are unfamiliar with the musical style succeed in detecting phrase boundaries? If phrase segmentation and boundaries primarily depended on subtle style-specific musical features, one would expect inexperienced listeners to score poorly, since they do not possess the implicit knowledge of the rules governing the style^50–52^. The hypothesis of Mungan et al.^23^ might therefore be accepted, that similar performance in segmentation by different groups of listeners indicates bottom-up processes rather than top-down, schematic knowledge. This in turn might lead to the supposition that boundary perception depends in part on cross-cultural features of music, such as those proposed in some recent studies^12,14,15^^,a.o.^, or of hierarchical structuring of musical rhythm converging into grouping^53^; and/or, finally, on universal cognitive capacities. The latter include Gestalt processes^23^, and implicit or incidental learning of style-specific musical features during exposure to music, which has been shown in cases of learning artificial musical grammars (even after very brief exposures)^54,55^ and learning modal features of Indian music^30^.

It is important to note that melodic or rhythmic schemata marking phrase and section endings may converge between different musical cultures, with regard to surface features (rests, expressive timing, slowing down, etc.) and melodic features, as argued, for instance, by Narmour^56,57^. Such convergence might have emerged historically from analogous social contexts and functions of music: both Indian and Western classical music have evolved as elaborate musical systems, performed for the delectation of attentive, knowledgeable connoisseurs in elite social contexts. Real-time articulation of structure to aid the listeners' immediate comprehension, and hence their experience of social engagement, might have particular importance in such music. Schematic similarities in different systems would then not imply universal characteristics of music. Further cross-cultural investigation of these questions will need to pay attention to ethnographic and historical contexts as well as to quantitative comparison of musical features.

## Supplementary Information

Supplementary Information

## Data Availability

All materials and analysis files associated with this study have been uploaded to the online repository at https://osf.io/khvmf.
