# Supplementary material for: Western listeners detect boundary hierarchy in Indian music: a segmentation study
Source: Sci Rep. 2021 Feb 4;11:3112. doi: 10.1038/s41598-021-82629-y (PMC7862587; doi:10.1038/s41598-021-82629-y)
Supplement: Supplementary file 1 — Supplementary Information [file 41598_2021_82629_MOESM1_ESM.docx]

Supplementary Materials for

**Western listeners detect boundary hierarchy in Indian music: a segmentation study**

Tudor Popescu^1,2,3,*^, Richard Widdess^4^, Martin Rohrmeier^1,5^

^1^ Institute for Art History and Musicology, Technische Universität Dresden, Germany

^2^ (present affiliation) Department of Cognitive Biology, University of Vienna, Austria

^3^ (present affiliation) Medical University of Vienna, Austria

^4^ Department of Music, School of Arts, SOAS University of London, UK

^5^ (present affiliation) Centre for Music and Science, École Polytechnique Fédérale de Lausanne (EPFL), Switzerland

* to whom correspondence should be addressed (tudor.popescu@univie.ac.at)

# Supplementary Methods

## Illustration of expert defined boundary regions (EDBRs)

Figure S1 is intended to illustrate the Level 1 and Level 2 (section-level and phrase-level) Expert Defined Boundary Regions, and the style of sitār ālāp.


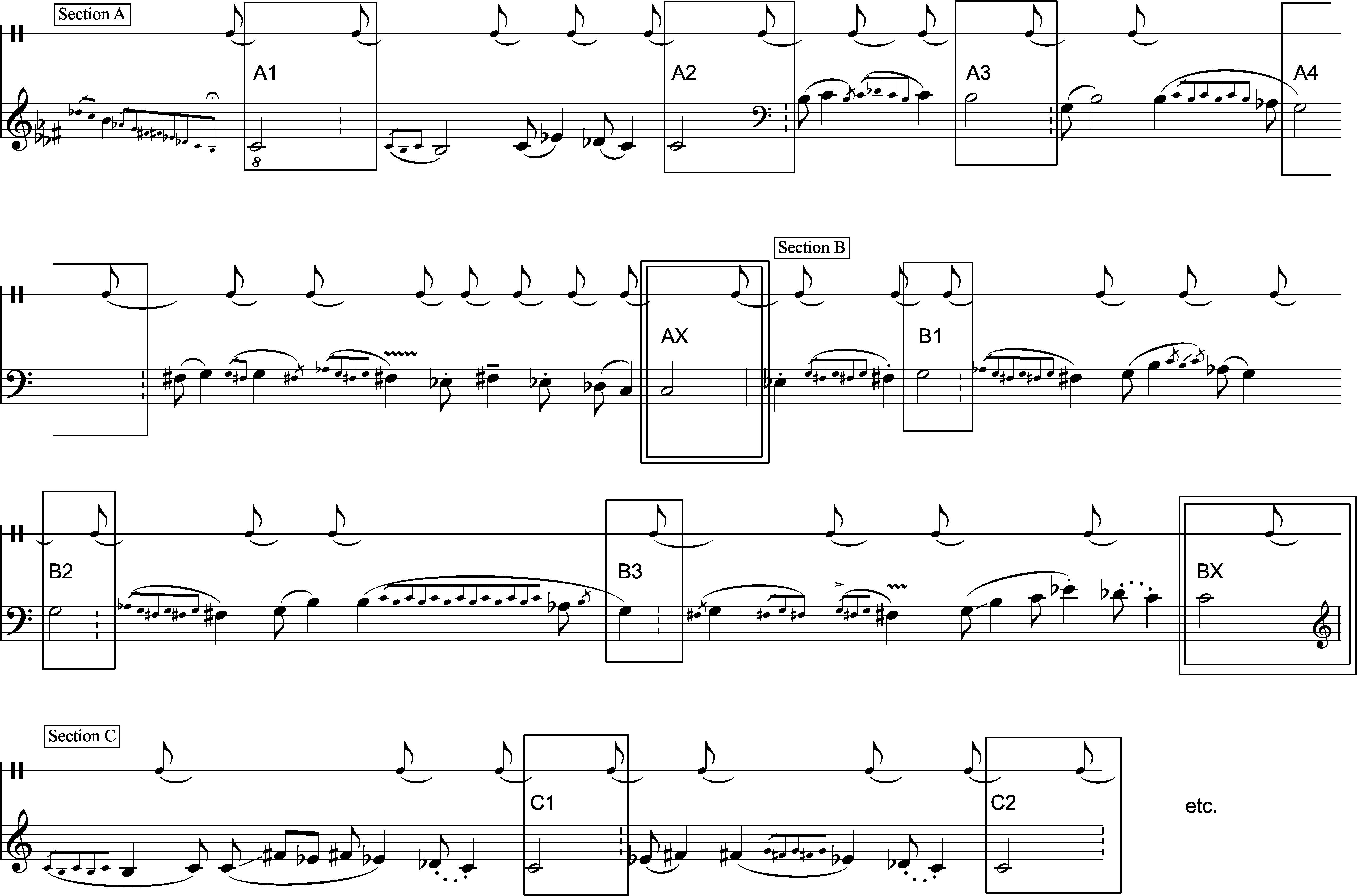


Figure S1: **Transcription of the ālāp in rāga Multānī** (beginning), showing the Expert-Defined Boundary Regions (EDBR). The double box marks boundaries at Level 1, single box Level 2; Level 1 boundaries are labelled AX, BX, and Level 2 as A1, A2 etc. X denotes the last boundary of a section. The last line shows the first two phrases of Section C. Because the rhythm is unmetered, durational values are relative, not proportional. The upper staff (single line) denotes plucks on the drone-strings of the sitar, tuned to 1 – 5 – 1’. The opening descending scale is played on the sympathetic strings (*tarab*), tuned to the scale employed in the rāga. A slur over a group of pitches indicates that only the first pitch is plucked, the others being played by pulling the string across the fret with the left-hand fingers. A dotted slur denotes a pull-off; a glissando line denotes sliding the stopping finger along the string, across one or more frets.

## Number of defined EDBRs

Because an original ecological recording was used, and given differences in the structures of the two rāgas, the number of boundaries could not be matched between the two rāgas, at either Level 1 or Level 2.

## Cross-validation of expert-defined segmentation

To validate our segmentation, we asked the sitarist who recorded the two rāgas, Dharambir Singh, to make an independent segmentation of both ālāps. While listening to his recordings, DS chose to discriminate three hierarchical levels of segmentation, which he referred to, using a language analogy, as "Paragraph", "Full Stop" and "Comma" boundaries. DS listened twice through each ālāp, pausing and replaying sections as required; each ālāp was then reviewed to confirm that the segmentation was satisfactory to him. DS was unaware of our existing segmentation, data or conclusions during this process.

The two segmentations are closely similar (for details see the document *RW-DS segmentation comparison.xlsx*, in the [online repository](https://osf.io/khvmf/?view_only=01f3755902994561a1c83b01702a859a)). Every boundary identified by author RW was also considered a boundary by DS . As can be seen from Table S1, the main differences are:

1. Because DS chose to identify three levels of boundary, he specified more boundaries (91) than RW did (51). The majority of additional boundaries, not corresponding to any of our boundaries, are Comma boundaries (36 out of 40). Only four Full Stop boundaries, and no Paragraph boundaries, are additional to our boundaries.

2. In a significant number of cases, DS and RW assigned different, but adjacent, hierarchical levels to a boundary. Thus 2 of our 9 Level 1 boundaries correspond to Full Stop rather than Paragraph boundaries in DS’s segmentation, but none to Comma boundaries. Our Level 2 boundaries correspond to Paragraph, Full Stop or Comma boundaries, but with a majority corresponding to Full Stop boundaries (as shown in Table S1).

These discrepancies can be explained in terms of the different criteria applied by DS and by RW in defining hierarchical levels: DS and RW did not agree such criteria in advance. For example, RW included main arrivals at pitch 1, in any octave, as section-level boundaries, but DS did not do so for 1 in the low octave. On the other hand he chose to regard the main arrival at pitch 5, in the middle octave, as a section-level boundary in Multānī, whereas RW only included arrivals at 1.

We conclude that the two segmentations are consistent – with each other (as qualitatively argued above), and with our participants (as quantitatively argued in the main text) – as to the *location* of boundaries at the two highest levels. They are inconsistent as to the *number* of hierarchical levels, and to the levels assigned to individual boundaries. There is however a correlation of levels that is maintained in 70 out of 91 cases (77%: see shaded values in Table S1): DS Paragraph = RW Level 1, DS Full Stop = RW Level 2, DS Comma = RW no boundary. DS’s third hierarchical level (Comma) includes surface-level (interstitial) groupings that our main segmentation (RW's) deliberately did not take into account; this information can be considered on another occasion.

In view of this strong support from the most authoritative possible source – the performer himself – and in view of the known criteria by which they had been defined, we decided to retain the RW EDBRs unchanged.

Table S1: **Hierarchical segmentations** (by DS and RW); totals for Toṛī and Multānī are combined. Shaded values denote underlying correspondence of level attribution between RW and DS.

|  |  | RW | | | Total DS |
| --- | --- | --- | --- | --- | --- |
|  |  | Level 1 | Level 2 | No boundary |  |
| DS | Paragraph | 7 | 2 | 0 | 9 |
|  | Full Stop | 4 | 27 | 4 | 35 |
|  | Comma | 0 | 11 | 36 | 47 |
|  | No boundary | 0 | 0 | 0 | 0 |
| Total RW | | 11 | 40 | [40] | 91 |

## Promptness score

Figure S2 shows the relationship between EDBRs and the promptness scoring system. This score decreased linearly from 1 at the beginning of the EDBR to 0 at the end. Note that plucks on drone strings during the boundary region are ignored.

Figure S2: Depiction of the timespan of an EDBR (red box), against the background of a sound waveform panned to the region of a phrase boundary (lower panel). The promptness score (upper panel) decreases linearly within the EDBR.

## Signal detection theory aspects

### Definition of 'hits'

In standard signal detection theory, the hit rate (HR) is defined as the number of hits divided by the total number of "signal present" trials (in this case, total number of EDBRs). The false alarm rate (FAR) is defined as the number of false alarms (clicks outside EDBRs) divided by the total number of "signal absent", i.e. non-EDBR events. This latter measure is in our case non-definable, given that in our data set, recordings were not modelled note-wise. Therefore, in order to have a finite denominator, we instead defined FAR by dividing by the participant's total number of clicks for the current listening. Duplicate clicks did not count towards any of the two measures, in other words a hit meant "at least one click within an EDBR", and likewise a false alarm meant "at least one click within an interstitial region".

In the defining formula of d', z(x) represents standardisation of the x distribution with respect to n(0,1), the normal distribution of mean 0 and standard deviation 1. Thus, the higher a participant's d', the more his/her clicks converge with the EDBRs, i.e. they fall inside EDBRs rather than in interstitial space.

We should note that, of the four Signal Detection Theory metrics (hits, false alarms, misses and correct rejections), the present type of data (clicks in response to temporally unfolding stimulus) only allows definitions of the *hit* and the *false alarm* rates, both corresponding to "signal present" responses. In other words, it makes little sense to talk of a *correct rejection* rate and a *miss* rate, as to do so would imply taking the lack of a response (all the moments when a key is not pressed) to be a response ("signal absent") in itself, which is not how the task was construed.

Furthermore, we note that, to compare listeners' ability to identify hierarchical grouping, we computed the participant-wise HRs, separately for (across) Level 1 and Level 2 EDBRs. We note that a similar plot cannot be made to compare instead the sensitivity indices (*d'* values). This is because d' is computed from both the HR and the FAR, but by definition, a false alarm lies outside of the boundary window and therefore cannot be assigned to one or the other type of EDBR. For this reason, the only Signal Detection Theory metric that we can compute for Level 1 vs Level 2 boundaries is HR, while d' can only be computed at participant level, i.e. without providing separate measures for Levels 1 and 2.

Finally, as stated in the main text, while clicks falling immediately before or after the EDBR could be interpreted as anticipations or delayed responses respectively, these were nonetheless not counted as "hits". We are aware that reaction times may be up to 2 sec. in music with no clear pulse (Hartmann et al., 2016), but we wished to exclude responses influenced by the following phrase.

### Plots of click densities vs hit rates

The reader might ask why it is that Figures 2 and 3 (in the main article), depicting click densities and HRs respectively, do not entirely agree with respect to the central tendencies of the distributions (e.g., for Toṛī medians, Level 2>Level 1 in terms of click density, but Level 1>Level 2 in terms of HR). The explanation lies with how the dependent variable in each plot is constructed. The click densities are a time-normalised (i.e. divided by window length) sum of the click count for each region, whether it be an EDBR or interstitial space. They are thus a measure of inter-participant consensus at region level. A key pressed within an EDBR is by definition a hit, and the HR is defined by dividing each participant's number of hits by the number of boundaries defined for the piece. This does not take the boundary window length into account, such that longer boundaries do not get 'penalised' for the extended time they take up, by having their click count divided by a long duration, as happens for the density plots. In other words, in the density plots (Figure 2 in the main article) we average boundary-wise and in the HR plots (Figure 3 in the main article) participant-wise, before in each case aggregating the averages to produce the respective distribution summary (dot plot). Thus, while each plot type is obtained from the same raw data set, this data contributes differently to how the two plots are created - and the difference lies with what is being plotted (average number of hits per time window; versus average participant-wise HR) and the dimension along which we average (per window, or per participant). Also contributing to the incongruence between these plots is the fact that the HR plots correct for (i.e. ignore) duplicate clicks within a single boundary window; whereas the histograms count every click towards the total.

# Supplementary Results

## Moving-cursor videos (interactive versions of Figure 1)

The [project](https://osf.io/khvmf/?view_only=01f3755902994561a1c83b01702a859a) created within the OSF online repository contains videos that show, for each rāga, the real-time correspondence between the music as heard by the listeners, and the statistics of their clicks at each moment, as displayed in Figure 1 in the main article. The leading edge of the cursor approximates to the current time-point. We defined synchronisation points between the audio and the histogram manually at the beginning and end of each track, and the software (iAnalyse, Pierre Couprie Software) interpolated the cursor movement automatically between the two points. Any slight audio-video asynchrony in these videos is likely to be due to the manual synchronisation points.

## Correlation plots

Please see the main manuscript for cross-references to the results plotted in the figures below. All correlations are across participants.

Figure S3: **Correlation between promptness of response to Level 1 and 2 EDBRs**, for Toṛī and Multānī. Each participant’s promptness scores at Level 1 and Level 2 (see Figure 4 in the main article) are here represented by a single data point. A least-squares regression line is superimposed on the scatterplot, with the 95% confidence band around it shaded. Squares denote participants familiar with Indian music (n=3 for Toṛī, n=3 for Multānī). Values annotated in the plot represent Pearson's correlation coefficients. ***: p<.001

Figure S4: **Correlation between (standardised) musicianship scores and d'**, for Toṛī and Multānī. Data points represent individual participants’ scores, averaged across listenings. Squares denote participants familiar with Indian music (n=3 for Toṛī, n=3 for Multānī). Red lines in the marginal histograms denote medians.

Figure S5: **Correlation between (standardised) musicianship scores and promptness** to Level 1 (top) and Level 2 boundaries (bottom), for Toṛī (left) and Multānī (right). Data points represent individual participants’ scores, averaged across repetitions. Squares denote participants familiar with Indian music (n=3 for Toṛī, n=3 for Multānī).

Figure S6: **Correlations between first and second listenings (repetitions)**, in terms of the HR, FAR, d', and promptness scores of all N=65 participants. The correlation statistics reported pooled data across both levels (if applicable: i.e., for HR and for promptness).

Also relevant for the question of inter-listening differences, the mean promptness score has decreased from the first to the second listening, both for Tori (1.75 to 1.59; paired-samples *t*-test: CI for *t* = [-0.01, 0.33], *p* = 0.069) and – significantly so – for Multānī (3.20 to 2.72; CI = [0.11, 0.84], *p* = 0.011).

Figure S7: **Correlation between HRs and average promptness per hit** (total promptness score divided by the number of hits), pooling across the two levels and the two repetitions. The relationships are very similar when d' is plotted on the x axis.

Figure S8: **Correlation between FARs and average promptness per hit** (total promptness score divided by the number of hits). The non-significance of these correlations means high promptness scores cannot be arrived at merely by a response strategy biased towards indiscriminate clicking (leading to a high FAR).

## Distribution plots

Figure S9: **Distribution of click densities across participants**, obtained by dividing the total number of clicks in each region type by the summed duration of regions of that type (in seconds), across both listenings. Note that, unlike Figure 2 in the main manuscript, here dots represent participants rather than regions.

# Supplementary References

Hartmann, M., Lartillot, O., & Toiviainen, P. (2016). Multi-scale Modelling of Segmentation. *Music Perception: An Interdisciplinary Journal*, *34*(2), 192–217. https://doi.org/10.1525/mp.2016.34.2.192
